# Exocentric coding of the mapping between valence and regions of space: Implications for embodied cognition

**DOI:** 10.1016/j.actpsy.2021.103264

**Published:** 2021-03

**Authors:** Adele M. Pacini, Philip J. Barnard

**Affiliations:** a The Open University, Walton Hall, Milton Keynes, MK7 6AA, United Kingdom of Great Britain and Northern Ireland; b MRC Cognition and Brain Sciences Unit, 15 Chaucer Road, Cambridge, CB2 7EF, United Kingdom of Great Britain and Northern Ireland

**Keywords:** Embodied cognition, Spatial encoding, Exocentric, Egocentric

## Abstract

Converging evidence has established that positive concepts presented on a computer screen are associated with upper regions of space, and negative concepts with a lower region of space. One explanation for this is that understanding positive or negative concepts requires the re-experiencing of direction, whereby “happy is up” and “sad is down.” However, it is unclear how the regions of space are encoded in these paradigms, space can be encoded in relation to oneself (egocentrically) or in object centred coordinates that are independent of oneself (exocentrically). The current study compares exocentric and egocentric coding of space, using a variation of the Meier and Robinson (2004) paradigm. Participants were asked to evaluate valenced concepts in either the upper or lower half of the screen. Spatial primes were used such that the concepts were preceded by either an upwards or a downwards eye movement. Exocentric coding of space in this paradigm was the computer screen, whilst egocentric coding was the eye movement used to access the top or bottom of the screen. It was proposed that egocentric coding of space, being coded in the body, provides evidence of a stronger relationship between the original bodily state of ‘up’ or ‘down’ and subsequent simulation. However, significant results supported an exocentric coding of space, with faster responses to positive concepts in the upper half of the screen, and to negative concepts in the lower half, irrespective of the direction of the eye movement preceding it. The implications of this for embodied cognition are discussed.

## 1 Introduction

The relationship between conceptual processing and regions of space has generated a considerable amount of research interest (Globig et al., 2019; Meier & Robinson, 2004; Sasaki et al., 2016). In a seminal study, Meier and Robinson (2004) showed that the evaluation of positive words was faster when they were presented in the upper half of a computer screen, and negative words evaluated faster in the lower half. This finding has since been replicated and extended to consider the relationship between body movements and valenced concepts. For example, Gozli et al. (2013) measured eye movements in response to a valence evaluation task where the upper and lower regions of the screen were irrelevant to the task. Following an evaluation of positive concepts, eye movements deviated towards the upper region of the screen when responding to a target on either the left or right of the screen.

The explanation for these congruency effects is that we simulate sensorimotor representations in concrete and abstract conceptual processing (Barsalou, 1999; Lakoff & Johnson, 1999). Barsalou's (1999) Perceptual Symbol Systems (PSS) approach claims that the evaluation of stimuli activates simulations of sensorimotor representations, which would be active in the actual perception of the stimuli. Our knowledge about a concept like “bird” would comprise of simulations of looking up at the sky and seeing the bird fly. For abstract concepts of valence, positive and negative concepts are thought to be associated with regions of visual space because evaluations are made on the basis of physical metaphors (Lakoff & Johnson, 1980; Pecher et al., 2011; Winter et al., 2015). The physical metaphors in this case are spatial - positive concepts being related to upper regions of space (e.g. she was over the moon, he was jumping with joy) and negative concepts with lower regions of space (e.g. he was down in the dumps, she was sinking in to a depression, see Lakoff & Johnson, 1999). Whilst the relationship between valence and regions of space is clearly reflected in language, the representation of it within an embodied framework is less transparent. Physical objects in the environment, like “bird” or “fish” are located externally to the body, typically within either an upper or lower region of space. However, the physical metaphors outlined above are linguistic analogies of an internally felt sensorimotor state, for instance the coding of “down” when feeling sad manifests in a lowered eye gaze, hunched posture, and a phenomenological experience of feeling “low.” Given the congruency effects observed between valence and regions of space, how this effect relates to sensorimotor simulations is not yet clear.

In addition, methodological differences such as the timing of the stimuli, whether the concept is abstract or concrete, and the task instructions, may all impact on the nature of the spatial congruency effects observed. Inhibitory rather than facilitatory effects have been observed in some studies (Bergen et al., 2007; Richardson et al., 2003). For example, Estes et al. (2008) found that response times to a target letter were slower if a previously presented word pair matched the typical location of the word pair (e.g. presenting head at the top of the screen, or foot at the bottom of the screen) than if it mismatched The timing of the stimuli appears to account for these inhibitory findings whereby up/down words tend to interfere with targets in compatible locations in discrimination tasks when targets appear within 400 ms after the word, whereas facilitation is observed in detection tasks when the time between words and targets is longer than 400 ms or when semantically related words and targets are used (Gozli et al., 2013).

Other researchers have argued that their results reflect a congruency between simulation of individual words meaning and spatial position (Pacini & Barnard, 2011; Richardson et al., 2003; Setic & Domijan, 2007); whilst some studies point to a congruency between a simulation of task relevant location and spatial attention (Pecher et al., 2010) rather than word specific simulations. Taken as a whole, the results from this literature suggest that the mechanisms underpinning spatial congruency effects are not yet fully understood.

The impact of methodological factors on the nature of the congruency effects observed, in addition to questions about the relationship between concepts and regions of space, both suggest that the role of the task environment bears closer scrutiny. In all of these tasks, as in much cognitive research, stimuli were presented on a computer screen. The question posed by the current study is whether the stability of the visual space provided by the computer screen, determines the nature of the mapping between concepts and the coding of regions of space. In doing so it contributes to a body of research concerned with the further specification of processes used by simulation mechanisms (Petilli et al., in press; Pezzulo et al., 2011). As Ostarek and Huettig (2019) note, unpacking the task dependency of embodied processing is an important issue if we are to move beyond establishing the existence of embodied simulations and look at their importance in routine cognitive processing.

Using a variation of a paradigm developed by Meier and Robinson (2004), the current study compares alternative processes used in the coding of visual space, in relation to captured modal states for valenced concepts.

With regard to the simulation of modal states in abstract conceptual processing, Meier and Robinson (2004) offer two different explanations for the congruency effects observed in their studies. Where valenced words are presented either in the top or the bottom of the screen, evaluation is facilitated in the congruent condition because the spatial metaphor is consistent with the actual position of the word. Where valenced words are presented in the centre of the screen and followed by a spatial probe at the top or the bottom of the screen, it is suggested that the evaluation of valence activates areas of visual space. These explanations suggest a bi-directional effect between location and valence evaluations, whereby the action of attending to a congruent location in the screen facilitates evaluations, and the process of evaluating valence primes attention to the congruent location. For both of these explanations, it is unclear how this relates to the original modal states captured in perceptual or motoric regions of the brain. If conceptual evaluation does activate regions of visual space, then there are two alternative ways of coding this, one is body centred, or egocentric, the other is exocentric where the space occupied by the word uses external points of reference to encode location (Paillard, 2005). A stronger embodied case could be made if the perceptual frame of reference was coded in the body, as simulations of “up” or “down”, as most motor actions, would be expected to use a body centred frame of reference. With this alternative, there would be a relatively direct mapping between the original capturing of the multimodal state, and the subsequent simulation of it. This would be consistent with research showing congruency effects between proprioceptive feedback and affective states or judgements (for a review see Niedenthal et al., 2005). Whilst other areas of the body (e.g. head, trunk, arm, leg) may be used as the egocentric reference, the clearest action component in this task is the eye movements used to locate the word or spatial probe. In terms of the original sensorimotor states, this is grounded in a lowered eye gaze when feeling sad, and a raised eye gaze when feeling happy. Thus, if simulations of pre-existing sensorimotor states are activated to evaluate valenced concepts, then this would result in faster eye movements when valence and direction of eye movement is compatible.

However, direction could also be coded exocentrically, relative to the structure of the visual background afforded by the computer screen. In this case, there would not be any effect of eye movement, simply one of word location. Relative to the centre of the computer screen, “up” would be the upper half of the screen, and “down” the lower half. Whether the eye movement used to access was up or down would be irrelevant, as the process would be based on exocentric spatial coding. A result in this direction would support embodiment in that cognitive representations of valence concepts are associated with representations of experiential states. However, the activation of up/down as a disembodied location would not necessitate a particularly strong embodied framework, the multimodal state which was captured originally need not be directly mapped on to a simulation of up/down, where up/down is coded relative to a computer screen. A related idea was forwarded by Boroditsky and Ramscar (2002) who argue that abstract knowledge is based on representations of sensorimotor domains, but that these are functionally separable from the representations directly involved in sensorimotor experience. That is, sensorimotor information may be activated in the processing of abstract knowledge, but not necessarily require the re-enactment of sensorimotor systems. Similarly, Estes et al. (2008) suggest that one locus for the spatial congruency effect is a non embodied association between concepts and spatial attention. Thus, the evaluation of valence may be associated with representations of up/down, but not necessarily activating any body centred simulations.

The current experiment alters the design of Meier and Robinson (2004), participants were asked to respond to a word in either the upper or lower region of space, however, the eye movement used to arrive at that location will vary. Specifically, words will be presented in either the upper or the lower half of the screen, with the eye movement used to arrive at the location being either upwards or downwards. Data consistent with an egocentric coding of space would show an interaction between eye movement and valence, whereby positive words are responded to faster following an upwards eye movement, and negative following a downwards eye movement, irrespective of whether the word appears in the upper or lower half of the screen. For exocentric coding of space, positive words will be responded to faster in the upper half of the screen, and negative words in the lower half of the screen, irrespective of the preceding eye movement.

## 2 Method

### 2.1 Participants

Based on the data from Meier and Robinson's (2004) Experiment 1, a minimum of 17 participants are required to have power of 80% to yield a statistically significant result, with a one-tailed test and significance level set at 0.05. Thus, a total of 25 participants (12 females) from the MRC Cognition and Brain Sciences Unit volunteer panel were tested. Participants had a mean age of 36 years (SD = 8 years). All participants were native English speakers, between 18 and 65 years of age, and reported no diagnosis of dyslexia in response to a screening question. Participants received an honorarium of £5 (approximately U.S $8 or 6 Euros) per hour for their participation in the project. The Cambridge Psychology (CPREC) research ethics committee approved the study.

### 2.2 Stimuli

Stimuli were those used in the original experiment by Meier and Robinson (2004). These were 100 positive and negative words, which had previously been matched for number of letters, and for absolute difference of valence rating. The positive words were as follows: active, agile, ambitious, baby, brave, candy, champion, clean, cordially, devotion, dream, earnest, ethical, faith, festival, garden, generous, genius, gentle, gracious, heaven, hero, justice, kiss, leisure, love, loyal, mature, mercy, neat, nurse, polite, power, pretty, prompt, radiant, reliable, righteous, satisfying, sensible, sincere, sleep, studious, sweet, talented, trust, truthful, victory, wise, and witty.

The negative words were as follows: aimless, argue, beggar, bitter, cancer, cheat, clumsy, crime, critical, crooked, crude, cruel, danger, dead, defeat, delay, devil, diseased, divorce, enemy, fickle, foolish, fraud, greedy, hostile, insane, insolent, liar, mediocre, mosquito, nasty, neurotic, obnoxious, poison, pompous, profane, rude, sarcastic, shallow, sloppy, sour, spider, steal, stingy, theft, touchy, ugly, unfair, vain, and vulgar.

### 2.3 Procedure

Participants were tested individually and gave their informed consent prior to testing. Consistent with Meier and Robinson's (2004) procedure there were no practice trials. To avoid an overt link between valence and location, participants were told that the study was investigating the impact of spatial attention on reaction times to words. Participants were instructed to pay attention to the spatial probes, which would appear in succession before the presentation of a valenced word. Both spatial probes and words were presented for 1000ms. On presentation of the word, they were asked to press “p” if the word was positive, and “q” if it was negative. “INCORRECT” appeared in red for 1000 ms if the evaluation was incorrect. Each word was presented twice (200 trials in total), in both the upper and lower half of the screen. The order of the words and condition were both randomised, the response keys were counterbalanced across participants. Each task type had 50 trials, these were split in to positive and negative words evenly. All trials were run in one experimental block, the entire experiment took approximately 20 minutes per participant.

**Fig. 1:**
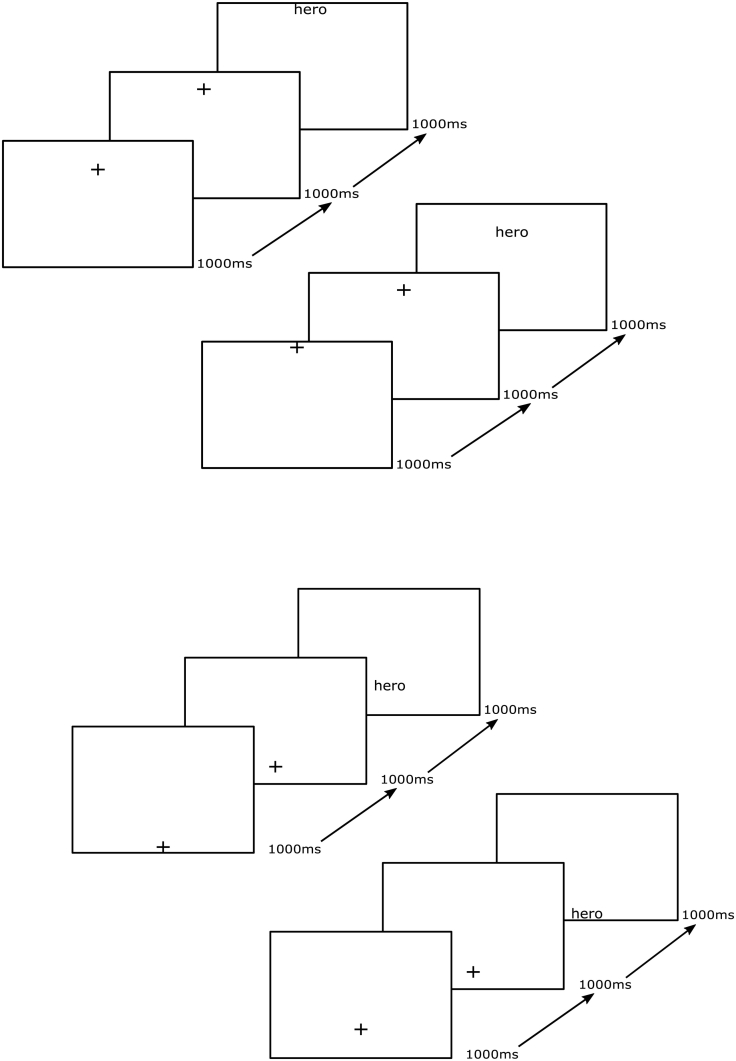
Schematics for each of the location x eye movement conditions, valence shown here is an example of a positive word. All words and fixation crosses were presented in the centre of the horizontal axis (in coordinate terms x = 50, on the y-axis, y = 0 was the top of the screen, y = 100 was the bottom.) The upper block of Fig. 1 shows the trials in the upper half of the screen. The top schematic shows the upwards eye movement condition, the first probe is at (50, 40) the second at (50, 30) and the word at (50, 20). The schematic beneath this shows the downwards eye movement condition the first probe is at (50, 0) the second (50, 10) and the word (50, 20). The lower block of Fig. 1 shows the trials in the lower half of the screen. The top schematic shows the upwards eye movement condition, first probe at (50, 100), the second (50, 90) and the word (50, 80). The bottom schematic shows the downwards eye movement the first probe was at (50, 60), the second (50, 70) and the word at (50, 80).

The experimental procedure is summarised in Fig. 1. All words and fixation crosses were presented in the centre of the horizontal axis (in coordinate terms x = 50, on the y-axis, y = 0 was the top of the screen, y = 100 was the bottom.), for each participant, the computer monitor was adjusted so that their level eye gaze was aligned with the centre of the screen. The upper block of Fig. 1 shows the trials in the upper half of the screen. The top schematic shows the upwards eye movement condition, the first probe is at (50, 40) the second at (50, 30) and the word at (50, 20). The schematic beneath this shows the downwards eye movement condition the first probe is at (50, 0) the second (50, 10) and the word (50, 20). The lower block of Fig. 1 shows the trials in the lower half of the screen. The top schematic shows the upwards eye movement condition, first probe at (50, 100), the second (50, 90) and the word (50, 80). The bottom schematic shows the downwards eye movement the first probe was at (50, 60), the second (50, 70) and the word at (50, 80).

## 3 Results

For comparability the data analysis procedure follows Meier and Robinson (2004) i.e., prior to analyses all reaction times, which were 2.5 SD above or below the grand latency mean were replaced with the 2.5 SD reaction time (these were, without exception, above the mean). Incorrect responses were removed, based on the intended valence of Meier and Robinson's (2004) stimulus set. On average 7% (SD = 1.3) of the data points were replaced or removed, per participant. Table 1 gives the means and standard deviations for response time for each response type.

**Table 1:** The means and standard deviations (in parentheses) for each response type.

	Positive		Negative	
	Top	Bottom	Top	Bottom
Upwards eye movement	831 (197)	827 (193)	877 (206)	838 (195)
Downwards eye movement	759 (152)	814 (161)	805 (175)	828 (179)

The results of the 2 (Eye movement, Up, Down) x 2 (Location, top, bottom) x 2 (Valence, positive, negative) is shown in Table 2.

**Table 2:** For the reaction times, a 2 (Valence: positive, negative) x 2 (Location: top, bottom) x 2 (Eye Movement: upwards, downwards) repeated measures ANOVA was carried out on the dependent variable response time (RT).

Source of variance	SS	MSE	F(1,23)	*p*-value	η^2^
Eye movement	83,545.67	1966.94	42.48	>0.001	0.65
Valence	41,284.86	1877.16	21.99	>0.001	0.49
Location	3676.20	3676.20	2.29	0.14	0.09
Location*eye movement	44,216.93	2406.68	18.37	>0.001	0.44
Location*valence	13,338.47	1264.26	10.55	0.004	0.31
Eye movement*valence	37.28	1655.68	0.02	0.88	0.001
Eye movement*valence*location	30.07	1798.87	0.02	0.90	0.001

The strongest effect was of Eye Movement, having a significant effect on response time, whereby downwards Eye Movements resulted in significantly faster responses to the stimulus than upwards ones. There was also a significant main effect of Valence, positive words being responded to faster than negative ones. The factor Location (top, bottom) was not significant. The Location x Eye Movement interaction was highly significant, this interaction was decomposed into two separate paired sample *t*-tests for the effect of Eye Movement in each location. In the top half of the screen, downwards eye movements were significantly faster t(23) = 7.34, *p* < .001, d = 0.43. In the bottom half of the screen there was no difference between upwards and downwards eye movements t(23) = 1.23, ns. There was a significant interaction between location and valence, which supports the Meier and Robinson hypothesis, this was decomposed in to two separate paired sample t-tests for the effect of location on the reaction time to positive and negative words. For the positive words, responses were significantly faster in the top half of the screen t(23) = 5.59, p < .001, d = 0.98. Negative words were responded to significantly faster in the bottom half of the screen, t(23) = 4.24, p < .001, d = 0.97. However, there was no significant interaction between Eye movement x Valence or Eye movement x Valence. Two large main effects were observed for eye movement and valence, neither of which was relevant to the interaction hypotheses. Therefore, we used the procedures of Rosnow and Rosenthal (1989) to examine interactive effects after the removal of the main effects for eye movement and valence, i.e. residual means. Residual means are calculated by removing the main effects of the experimental treatments and the grand mean of the dependent variable from the group means.

**Fig. 2:**
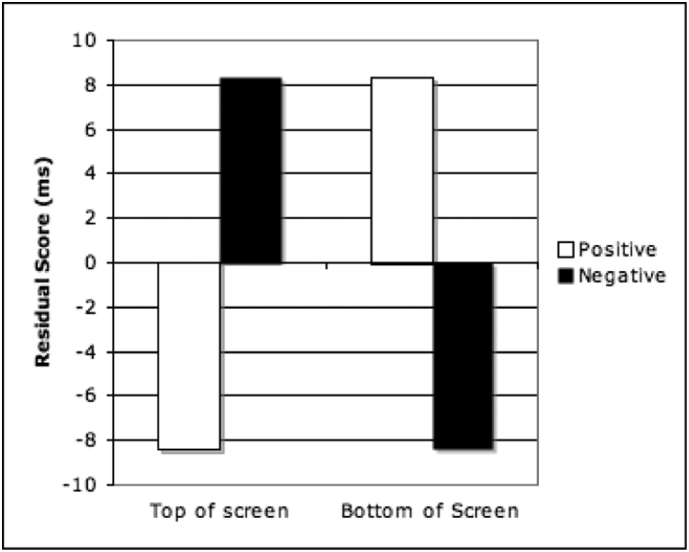
Residual scores for the Valence x Location interaction.

As shown in Fig. 2, these residual scores revealed that participants were faster to categorize positive words when presented on the top of the screen, but faster to categorize negative words presented on the bottom of the screen, after removal of the main effect of valence.

## 4 Discussion

The results from this experiment suggest that it is the exocentrically coded top/bottom of the computer screen, which is congruent with valence, rather than the motoric eye movement itself. There was a significant interaction between Location and Valence, positive words were responded to faster in the top half of the screen than the bottom half of the screen; conversely, negative words were responded to faster in the bottom half of the screen and slower in the top half of the screen. This pattern of results was consistent across raw and residual means (Table 1 and Fig. 2). However, there was no interaction between Eye Movement and Valence. This result is inconsistent with research showing congruency effects between proprioceptive feedback and affective states or judgements (e.g. Nair et al., 2015; Strack et al., 1988). We suggest that our findings diverge due to the nature of the task, in both Nair et al. (2015) and Strack et al. (1988) the study design altered participants' proprioceptive state. In our study this was an optional response to presented stimuli. The data obtained in the current study argues against a strong sensorimotor simulation account. Bodily action in this case appears to be irrelevant, the effect depends on the location of the word, relative to the computer screen.

This pattern of data can be explained by reference to the demands of the task, and to types of spatial encoding. Paillard (1987) argues for two types of encoding, egocentric or “sensorimotor” and exocentric or “representational”. These ways of processing spatial information are not mutually exclusive, and the distinctions between them explains why this particular task uses a representational model of space.

Egocentric processing of space is ideally suited to the active exploration of the environment. The body has various sites (e.g. hands, eyes, mouth) which delineate an autonomous sensorimotor space. These are delineated by both the perimeter of the receptive field of the sensory surface and the limits on its exploratory motility imposed by biomechanical characteristics. Briefly, the assumption is that motor activities modify the spatial relationships between a given sensory receptive field (visual, tactile, auditory, proprioceptive) and sensory targets located within the physical surrounding. They therefore provide the information required for the neural registering of proprioceptive information from orienting movements together with information about the position of targets within the sensory map of the receptive surface. Thus, this system provides an online, dynamic process by which active exploration provides a rapidly updated sensorimotor map.

However, there is also substantial evidence for an exocentric representation of environmental space. As Oakley (1985) argues, these exocentric representations provide “a safe means of problem solving during a period of behavioural silence”. This feature of the representational mode of spatial processing becomes apparent if we consider perceiving the stability of visual space. This has direct relevance to the current paradigm as the task is centred on the clearly delineated space of a computer screen. The cognitively based system uses retinal information to judge target position on the basis of cues from the structured visual array. This leads to a “hyper stability” of visual space this is directly dependent on attentional processes.

Thus it is likely that in using a computer screen as the visual environment, this task predisposes participants to use a cognitive rather than a sensorimotor process. Furthermore, it may be that in presented the concepts in English rather than other potentially more embodied languages (for example see Ghandhari et al., 2020) any sensorimotor activation is weakened further. In terms of the original multimodal state envisaged by Barsalou (2008), there is only a weak relationship between the original egocentric state of up/down captured throughout the brain's association areas, and the resulting activation of up/down coded exocentrically relative to a computer screen. If an embodied account is maintained in explanation of this finding, it points to a gap in current theory, which can account for the processes which occur between the simulation of an original multimodal state, and the execution of action which is strongly dependent on the task environment. It may be that the pure “embodied” component of this process occurs early in processing and is relatively small in comparison to subsequent symbolic processes. The findings from the current experiment do not necessarily undermine embodied frameworks, however, they do suggest that a stronger embodied theory where there is a direct relationship between original modal states and sensorimotor simulations, is not tenable in this case.

A further implication arises from the distinctions between egocentric and exocentric processing of space. Essentially this paradigm used a highly stable visual space, with little scope for active perception on the part of the participant. This kind of environment is ideally suited to an exocentric representation of space, rather than constant updating through an egocentric mechanism. This raises the question of whether valence would naturally activate an exocentric representation of a region of space, or whether this is simply an artefact of the task. Indeed, Fini et al. (2015) compared an avatar and an inanimate object in a virtual reality to determine whether our perception of near/far is influenced by the action potential of the object used. They found that participants perceived a beach umbrella in the scene to be nearer in relation to an avatar than an inanimate object. The authors conclude that we simulate the action potential of objects in our environment to aid our perceptual judgements. Certainly, in a richer environment, for example social interaction, it would make more sense for a positive phrase to activate egocentrically coded postural adjustments, potentially also mirroring the interlocutor, than activating a region of space. Taking these findings together, our experiment does not support the idea that strongly embodied processes (i.e. egocentric) necessarily mediate the mapping between conceptual information and sensorimotor information. Rather, the structure of the environment determines what type of process is used.

## Declaration of competing interest

None.
